# Quantifying Anharmonic Vibrations in Thermoelectric Layered Cobaltites and Their Role in Suppressing Thermal Conductivity

**DOI:** 10.1038/s41598-018-29259-z

**Published:** 2018-07-24

**Authors:** Susumu Fujii, Masato Yoshiya, Craig A. J. Fisher

**Affiliations:** 1 Department of Adaptive Machine Systems, Osaka University, 2-1 Yamadaoka, Suita, Osaka 565-0871 Japan; 2 Nanostructures Research Laboratory, Japan Fine Ceramics Center, 2-4-1 Mutsuno, Atsuta, Nagoya 456-8587 Japan

## Abstract

Optimizing multiple materials properties which are simultaneously in competition with each other is one of the chief challenges in thermoelectric materials research. Introducing greater anharmonicity to vibrational modes is one strategy for suppressing phonon thermal transport in crystalline oxides without detrimentally affecting electronic conductivity, so that the overall thermoelectric efficiency can be improved. Based on perturbed molecular dynamics and associated numerical analyses, we show that CoO_2_ layers in layered cobaltite thermoelectrics Na_*x*_CoO_2_ and Ca_3_Co_4_O_9_ are responsible for most of the in-plane heat transport in these materials, and that the non-conducting intermediate layers in the two materials exhibit different kinds of anharmonicity. More importantly, thermal conduction is shown to be altered by modifying the structure of the intermediate layers. The simulation methods developed to quantify the effect of anharmonic atomic vibrations on thermal conductivity provide a new tool for the rational design of thermoelectric materials, and the insights gained should hasten the attainment of higher conversion efficiencies so that thermoelectrics can be put to widespread practical use.

## Introduction

Thermoelectric generators promise to enable efficient recovery of waste heat by converting it directly into electrical power. To be practicable, such devices must be made of thermoelectric materials with high conversion efficiencies, which are usually measured in terms of a figure of merit, $ZT={S}^{2}\sigma T/\kappa$, where *T* is temperature and *Z* is proportional to the square of Seebeck coefficient, *S*; electronic conductivity *σ*; and the inversely proportional to the thermal conductivity, *κ*. To attain a high *Z* value, a material ideally should have high *S*, high *σ* and low *κ*. However, in most classes of materials all three properties are strongly correlated and not easily decoupled^1^, so, for example, increasing the electronic conductivity is typically accompanied by an increase in thermal conductivity, making it notoriously difficult to obtain figures of merit high enough for practical application.

Recent studies have shown that introducing greater phonon anharmonicity to a structure is an effective means of suppressing phonon thermal conductivity, *κ*_ph_, the chief component of thermal conductivity in a solid, without compromising the electronic properties^2,3^. The complex relationship between phonon anharmonicity and thermal conductivity, however, has meant that a rigorous quantitative understanding of these phenomena is lacking for all but the simplest of materials. Examining vibrational anharmonicity and its effect on thermal conduction is thus an important step in the development of innovative strategies for improving the performance and efficiency of thermoelectric materials^4^.

Layered cobaltites such as Na_*x*_CoO_2_ and (Ca_2_CoO_3_)_0.62_CoO_2_ (normally approximated as Ca_3_Co_4_O_9_) contain no toxic elements and are examples of materials with high power factors, *S*^2^*σ*, and modest *κ*_ph_. The electronic properties of these materials have been measured using various experimental methods^5–8^, and it has been determined through a combination of experimental^9,10^, theoretical^11,12^ and *ab initio* modelling studies^13–16^ that the highly correlated *d* electrons in the CoO_2_ layers common to these cobaltites are responsible for the high *S* and *σ* values. Their modest *κ*_ph_ values, on the other hand, have mostly been attributed to the complicated layered structures that characterise the two compounds^8^. Recent experimental studies suggest that crystal layers adjacent to CoO_2_ layers, viz., Na layers in Na_*x*_CoO_2_ and Ca_2_CoO_3_ layers in Ca_3_Co_4_O_9_, play a role in suppressing *κ*_ph_, either directly or indirectly. In the case of Na_*x*_CoO_2_, Na vacancies have been reported to exhibit a rattling mode^17^_,_ while in misfit-layered Ca_3_Co_4_O_9_, atoms in the Ca_2_CoO_3_ layers have been reported to exhibit anomalously large static and thermal displacements^18–21^. In both cases the layers adjacent to CoO_2_ layers appear to enhance phonon scattering, although the exact mechanism by which they do this is not yet clear.

To address this issue, we analysed atom vibrations and phonon transport mechanisms in these compounds quantitatively using the perturbed molecular dynamics (MD) method^22^ in conjunction with lattice dynamics (LD) simulations, focussing on the degree and distribution of vibrational anharmonicity in different layers. The role of anharmonic vibrations in the non-conducting layers in suppressing thermal conductivity was quantified by performing real-space and modal (or frequency) analyses of phonon thermal conductivities in each layer type in Na_*x*_CoO_2_ and Ca_3_Co_4_O_9_. The results revealed that, although most of the thermal energy is transported by the CoO_2_ layers, phonon thermal conduction can be suppressed by increasing the vibrational anharmonicity in the layers adjacent to the CoO_2_ layers. Differences in how the Na layers and Ca_2_CoO_3_ layers interfere with the phonon transport were also identified. From these insights it appears that further increases in anharmonicity in layered cobaltites and related classes of materials through tailoring of the non-conducting layers can result in increased *ZT* values and hence improved thermoelectric performance.

## Intralayer Thermal Conduction

Table 1 reports overall thermal conductivities of Na_*x*_CoO_2_ (*x* = 1.0, 0.5) and Ca_3_Co_4_O_9_ at 300 K in each crystallographic direction obtained from MD simulations. The out-of-plane thermal conductivities are lower than in-plane conductivities for all compounds, and the in-plane thermal conductivity decreases with decreasing Na content from NaCoO_2_ to Na_0.5_CoO_2_, with an even lower value in the case of Ca_3_Co_4_O_9_. These trends are in good agreement with experimental reports^23–26^. For example, the average of the calculated in-plane thermal conductivities of NaCoO_2_ at 300 K is 43.97 W/mK, which is close to the experimentally measured in-plane thermal conductivity reported for Na_*x*_CoO_2_ (*x* ≈ 1) of 37 W/mK^23^. In Na_0.5_CoO_2_, the average in-plane thermal conductivity at 300 K is 15.40 W/mK, which also compares well with the experimentally reported value of 16.5 W/mK (estimated using the Wiedemann-Franz law) for phonon thermal conductivity in single crystalline Na_*x*_CoO_2_^24^. In the case of Ca_3_Co_4_O_9_, anisotropic phonon thermal conductivity has been reported to be 4.5 and 2.1 W/mK in the in-plane and out-of-plane directions, respectively^26^, similar to values in this study of 6.92 and 3.32 W/mK in the corresponding directions. The good quantitative agreement gives us confidence that we can meaningfully examine the mechanism of phonon thermal conduction suppression in these materials using our classical MD model. Also, because electrons in layered cobaltites are transported within CoO_2_ layers^24,26^, in-plane phonon conduction is the most relevant with respect to optimising the thermoelectric conversion efficiency, so from here on we mainly focus on this aspect of phonon transport.

**Table 1:** Overall, partial and layer phonon thermal conductivities for Na_*x*_CoO_2_ (*x* = 1.0, 0.5) and Ca_3_Co_4_O_9_ at 300 K.

	NaCoO_2_			Na_0.5_CoO_2_			Ca_3_Co_4_O_9_		
	*X*	*Y*	*Z*	*X*	*Y*	*Z*	*X*	*Y*	*Z*
${\kappa }_{{\rm{overall}}}$	44.55 ± 1.67	43.39 ± 1.92	19.01 ± 1.37	16.69 ± 0.19	14.11 ± 0.49	3.71 ± 0.05	6.60 ± 0.15	7.24 ± 0.28	3.32 ± 0.58
${\kappa }_{{{\rm{CoO}}}_{2}}$	38.23	36.84	14.82	16.33	13.80	3.31	4.67	5.11	1.47
${\kappa }_{{\rm{adj}}}$	6.32	6.56	4.20	0.37	0.32	0.41	1.93	2.13	1.85
${\kappa \text{'}}_{{{\rm{CoO}}}_{2}}$	57.07	54.99	22.12	25.92	21.90	5.25	13.21	14.46	4.16
${\kappa \text{'}}_{{\rm{adj}}}$	19.14	19.86	12.71	0.99	0.85	1.10	2.99	3.29	2.86

Directions *X*, *Y* and *Z* refer to the Cartesian coordinate system, with *X* parallel to the *a* axis, *Y* parallel to the *b* axis, and *Z* perpendicular to the *ab* plane (and CoO_2_ layers); “adj” stands for layers adjacent to CoO_2_ layers.

Table 1 also summarises the calculated thermal conductivities for each type of layer. The partial thermal conductivities are a measure of the contribution of each type of layer to the overall thermal conductivity, which is defined here as an extensive variable such that

$${\kappa }_{{\rm{overall}}}={\kappa }_{{{\rm{CoO}}}_{2}}+{\kappa }_{{\rm{adj}}},$$

where ${\kappa }_{{{\rm{CoO}}}_{2}}$ and ${\kappa }_{{\rm{adj}}}$ are the partial thermal conductivities of CoO_2_ layers and the layers adjacent to them, respectively. The partial thermal conductivity of a particular layer depends on the volume ratio of the two types of layer, so to enable each type of layer’s thermal conduction to be compared on the same basis we also define “layer” thermal conductivities ${\kappa }_{{{\rm{CoO}}}_{2}}^{\text{'}}$ and ${\kappa }_{{\rm{adj}}}^{\text{'}}$ to be the partial conductivities normalised by layer thickness (see Methods section for details).

The results in Table 1 show that CoO_2_ layers exhibit a larger partial thermal conductivity than the adjacent layers in each of the three compounds. Even in Ca_3_Co_4_O_9_, in which the Ca_2_CoO_3_ layers are thicker than CoO_2_ layers, the latter account for more than 70% of ${\kappa }_{{\rm{overall}}}$. This indicates that in addition to electron transport, the CoO_2_ layers also transfer the majority of the heat energy through these materials. From Table 1 it can also be seen that the overall thermal conductivities of Na_0.5_CoO_2_ and Ca_3_Co_4_O_9_ are markedly lower than those of NaCoO_2_. Even more notable is the drop in layer thermal conductivities of CoO_2_ layers in Na_0.5_CoO_2_ and Ca_3_Co_4_O_9_ (by roughly 57% and 75%, respectively) compared to NaCoO_2_, which cannot be explained using a simple macroscopic model in which layer properties are independent of one another. The results thus indicate that increasing vibrational anharmonicity in the adjacent layers also lowers the thermal conductivity in the CoO_2_ layers, confirming the importance of the non-conducting layers in modifying the overall thermal conductivity.

**Figure 1:**
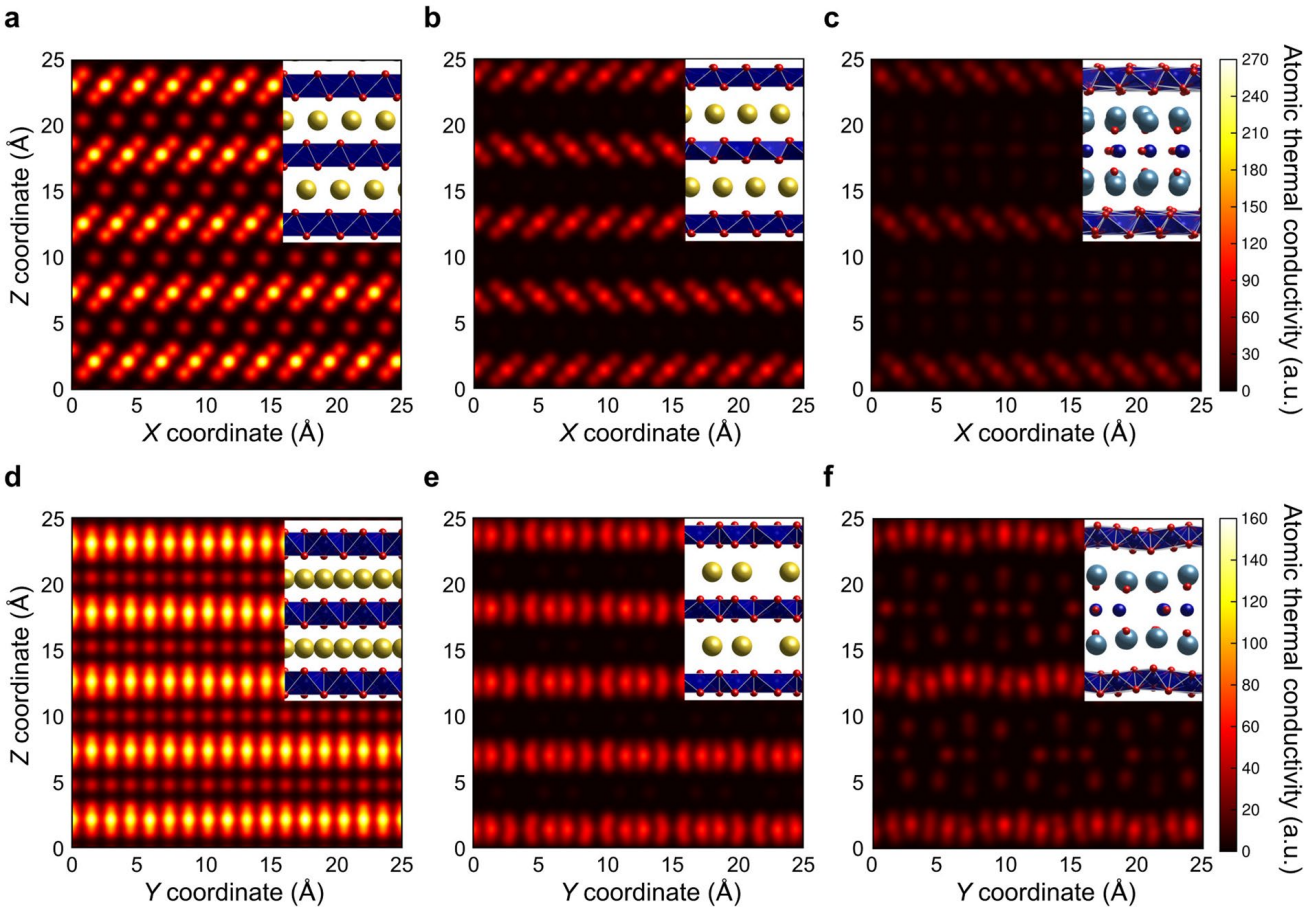
In-plane atomic thermal conductivities at 300 K. (**a–c**) Maps of *X* direction atomic thermal conductivities of **(a)** NaCoO_2_, **(b)** Na_0.5_CoO_2_, and **(c)** Ca_3_Co_4_O_9_ viewed down the *Y* direction. (**d–f**) Maps of *Y* direction atomic thermal conductivities of **(d)** NaCoO_2_, **(e)** Na_0.5_CoO_2_, and **(f)** Ca_3_Co_4_O_9_ viewed down the *X* direction. Note that the intensity range used in (**a–c**) is different to that used in (**d–f)**. Crystal structures corresponding to each map are shown as insets, with yellow, light blue, dark blue and red balls representing Na, Ca, Co and O ions, respectively^43^. Blue polyhedra represent edge-sharing CoO_6_ octahedral units.

The mechanism by which thermal conductivity is suppressed in the layered cobaltites can be gleaned better from the real-space analysis in Fig. 1, in which atomic thermal conductivities are visualised. Although phonon densities of CoO_2_ layers might be expected to be similar in each material at the same temperature, consistent with the results in Table 1, Fig. 1 shows that the partial thermal conductivities of these layers are strongly influenced by the type of layer sandwiched between the CoO_2_ layers, with the thicker but less heat-conductive rock-salt-type Ca_2_CoO_3_ layers more effective at suppressing ${\kappa }_{{{\rm{CoO}}}_{2}}$ than Na layers containing vacancies. Rébora *et al*. reported that the partial thermal conductivity of Ca_2_CoO_3_ layers is larger than that of CoO_2_ layers, which is the opposite of our results^27^. This is possibly because they used LD within the harmonic approximation and assumed the phonon relaxation time to be constant. In our MD simulations, anharmonic thermal vibrations were included intrinsically on account of the atomic interaction model used. This anharmonicity results in a much lower partial thermal conductivity in the Ca_2_CoO_3_ layers than CoO_2_ layers.

The mechanism by which Ca_2_CoO_3_ layers more effectively suppress phonon thermal conductivity is related to the incommensurate stacking of Ca_2_CoO_3_ and CoO_2_ layers in Ca_3_Co_4_O_9_. The lattice misfit introduces a strain that slightly displaces atoms in the CoO_2_ layers from their equilibrium lattice sites^18–21^, increasing the anharmonicity of their phonons. In earlier reports^28,29^ we demonstrated that atomic vibrations invoked by lattice misfit dominate suppression of $\kappa$ rather than static atomic displacements in the case of Ca_3_Co_4_O_9_. Although the structure and composition of Na layers in Na_*x*_CoO_2_ (*x* < 1) are very different to those of Ca_2_CoO_3_ layers in Ca_3_Co_4_O_9_, the misfit lattice strain appears to be the more effective mechanism for increasing phonon anharmonicity. To determine why this is so, we analysed the vibrational behaviour within layers adjacent to CoO_2_ in the three systems in greater detail.

## Anharmonic Vibrations in Layers Adjacent to CoO_2_ Layers

**Figure 2:**
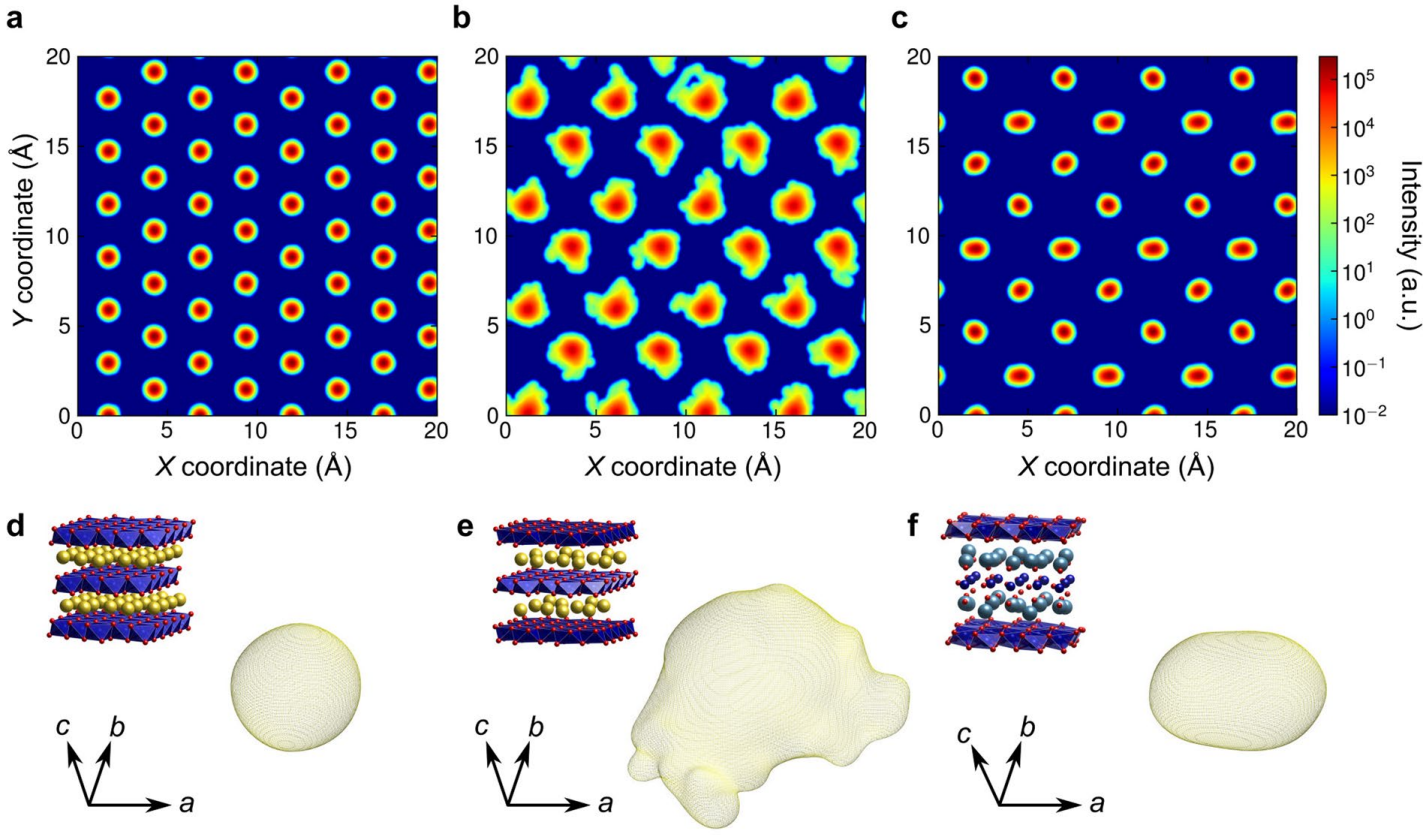
Vibrational density plots in layers adjacent to CoO_2_ layers. **(a–c)**, Density plots of atom vibrational motion within **(a)** a single Na layer of NaCoO_2_, **(b)** a single Na layer of Na_0.5_CoO_2_, and **(c)** a single Ca plane in a Ca_2_CoO_3_ layer of Ca_3_Co_4_O_9_, projected onto their *ab* (basal) planes. (**d–f**), Example of density plot isosurfaces of **(d)** a single Na ion in NaCoO_2_, **(e)** a single Na ion in Na_0.5_CoO_2_, and **(f)** a single Ca ion in Ca_3_Co_4_O_9_. In **d**, **e** and **f**, the crystal structure of each cobaltite is shown in the upper left hand corner.

Figure 2a shows a vibrational density plot for atoms in a single Na layer in NaCoO_2_ projected onto the basal plane together with a representative three-dimensional isosurface of the vibrational density of a single Na atom in that layer (Fig. 2d). These vibrational densities conform to the curvature of the potential energy hypersurface around each atom. All the projections are circular in shape as the vibrational densities in this case are spherical, indicating that Na atoms vibrate uniformally about their equilibrium lattice positions in the defect-free system. Figure 2b,e show corresponding results for Na_0.5_CoO_2_, in which each Na layer contains 50% vacancies. In contrast to the vacancy-free compound, the uneven isosurfaces in the case of Na atoms in Na_0.5_CoO_2_ reveal that their thermal vibrations are much more irregular and hence anharmonic.

Figures 2c,f show vibrational densities of Ca atoms in one Ca plane in one Ca_2_CoO_3_ layer of Ca_3_Co_4_O_9_ projected onto the basal plane together with a representative three-dimensional isosurface of one of the Ca atoms. The density distributions appear relatively uniform compared with those of Na ions in Na_0.5_CoO_2_. However, unlike in NaCoO_2_, there is considerable variety in the shapes of these isosurfaces, with some of them appearing distended in the *X* direction (ellipsoidal), even though the misfit direction is along the *Y* axis (see Supplementary Material Fig. S2 for details). This indicates that misfit between the two types of layers not only introduces a lattice strain but also causes the Ca atoms to vibrate anisotropically.

**Figure 3:**
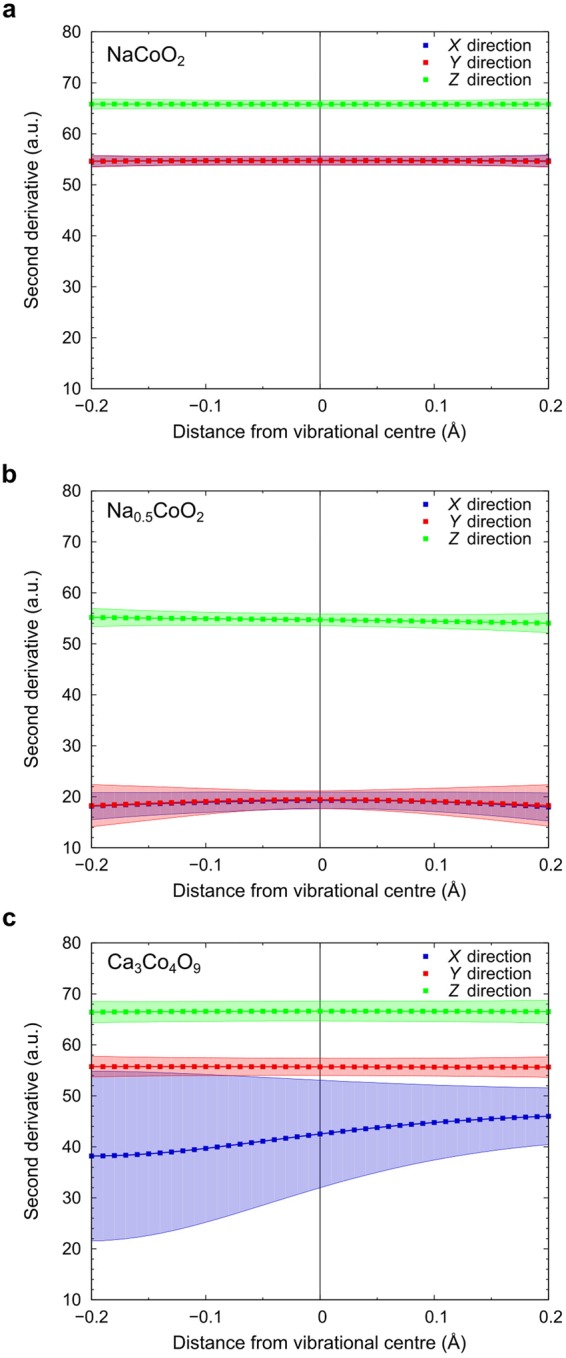
Potential energy second derivatives for cations in layers adjacent to CoO_2_ layers. Mean potential energy second derivatives (proportional to force constants) and associated standard deviations (shaded regions) for **(a)** Na ions in NaCoO_2_, **(b)** Na ions in Na_0.5_CoO_2_, and **(c)** Ca ions in Ca_3_Co_4_O_9_ in in-plane (*X* and *Y*) and out-of-plane (*Z*) directions.

The intensities of the vibrational density plots are a response to the potential energy fields experienced by the atoms, and thus, within the harmonic approximation, the second derivative of the intensities is proportional to the atomic vibrational force constant. Figures 3a,b show the average second derivatives calculated for Na ions in NaCoO_2_ and Na_0.5_CoO_2_, respectively, in the three principal directions as a function of distance from the equilibrium lattice position from −0.2 Å to 0.2 Å. When Na layers are vacancy-free, the second derivative is almost the same for all atoms with a narrow standard deviation. This means that, on average, the forces exerted on an Na atom by its neighbours are proportional to its distance from its equilibrium lattice position, i.e., Na vibrations in NaCoO_2_ are close to being harmonic. Highly harmonic atom vibrations explain the high phonon thermal conductivity of NaCoO_2_. In the case of Na_0.5_CoO_2_, the average second derivative of the Na vibrational density isosurface in the in-plane directions is approximately one third that of Na atoms in NaCoO_2_. Moreover, the further from the equilibrium lattice position, the larger the standard deviation, indicative of greater anharmonicity. Atoms exhibiting this kind of vibration are often described as “rattlers”, and their irregular thermal motion is associated with increased phonon scattering and hence lower phonon thermal conductivity^17^.

The average second derivatives of the isosurfaces of Ca ions in Ca_3_Co_4_O_9_ in each principal direction are plotted in Fig. 3c. In the *X* direction, the second derivative is highly asymmetric around the equilibrium lattice position and has a much larger standard deviation than in the other two directions (corresponding to large differences in the potential energy surfaces for *X* direction as shown in Fig. 2c). The vibrational motion in this direction is thus further from harmonic. Indeed, all atoms in Ca_2_CoO_3_ layers exhibit highly asymmetric vibrations. This irregular vibrational motion disturbs the motions of atoms in neighbouring CoO_2_ layers, resulting in a lower phonon thermal conductivity for Ca_3_Co_4_O_9_ even without introduction of point defects. Linking specific atomic vibration modes observed during MD with phonon modes calculated by lattice dynamics methods can provide further insights into thermal conductivity mechanisms and origins of anharmonicity, although for complex systems such as the cobaltites this is not always straightforward. In the case of Ca_3_Co_4_O_9_, the anisotropic decrease in the average of the second derivatives of the potential energy surface in the negative *X* direction can be explained in terms of low-frequency floppy modes within the Ca_2_CoO_3_ layers. This approach is discussed in more detail in section S2 of Supplementary Material.

Our analyses show that the anharmonic vibrations in Na_0.5_CoO_2_ and Ca_3_Co_4_O_9_ are very different to each other as a result of the different types of non-conducting layers separating the CoO_2_ layers. In Na_0.5_CoO_2_, the in-plane force constants of Na atoms (corresponding to second derivatives) are small but almost constant. Because of the small force constants, Na atoms can vibrate freely to some extent, resulting in uneven potential energy surfaces and higher standard deviations from equilibrium lattice positions compared to NaCoO_2_. In contrast, in Ca_3_Co_4_O_9_ the in-plane force constants of Ca atoms are comparatively large (similar in magnitude to those in NaCoO_2_) but asymmetric. The larger force constants in *Y* and *Z* directions constrain the vibrational densities to narrow bands, but the thermal vibrations in the *X* direction vary greatly, resulting in a large standard deviation from equilibrium lattice positions.

It is worth noting that our simulations may underestimate the degree of anharmonicity in these materials, since we employed simple two-body interatomic potentials that do not take into account subtle changes in electronic structure as bond distances lengthen and shorten during vibrational motion^30^. First-principles calculations may provide a more accurate picture of anharmonic vibration modes in these materials, but are much more computationally expensive. In real materials, atomic vibrations, and hence thermal conductivity, are also strongly affected by various crystalline defects, including impurity atoms, grain boundaries, surfaces, and so on, and thus can be expected to exhibit even greater variation than those observed in our model. Further experimental and computational work is needed to quantify the effects of crystalline defects on thermal conductivity in these layered cobaltites, including to what extent these features can be used to lower the thermal conductivity further.

## Modal Analysis of Phonon Scattering

**Figure 4:**
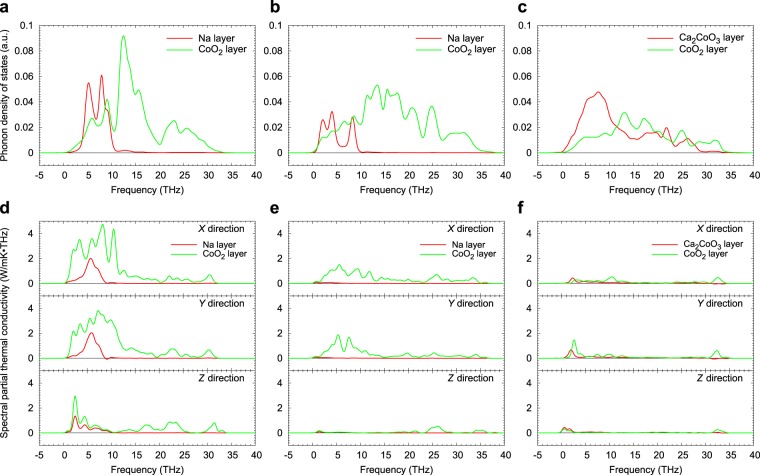
Phonon densities of states and spectral partial thermal conductivities at 300 K. **(a–c)**, Phonon density of states of layers in **(a)** NaCoO_2_, **(b)** Na_0.5_CoO_2_, and **(c)** Ca_3_Co_4_O_9_. (**d–f**), Frequency dependence of partial thermal conductivity of each type of layer in **(d)** NaCoO_2_, **(e)** Na_0.5_CoO_2_, and **(f)** Ca_3_Co_4_O_9_ for in-plane (*X* and *Y*) and out-of-plane (*Z*) directions.

**Figure 5:**
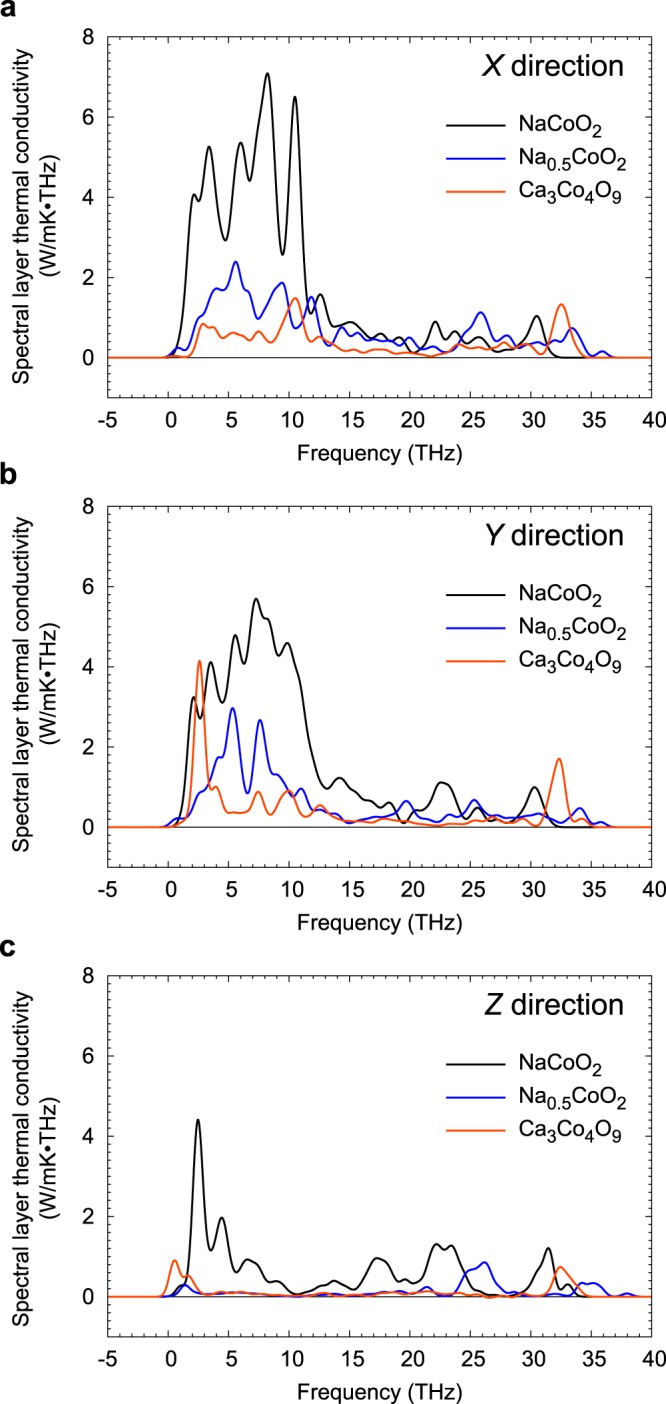
Frequency dependence of layer thermal conductivities of CoO_2_ layers. **(a–c)** Layer thermal conductivities of CoO_2_ layers in Na_*x*_CoO_2_ (*x* = 1.0, 0.5) and Ca_3_Co_4_O_9_ at 300 K in **(a)** *X*, **(b)** *Y*, and **(c)** *Z* directions as a function of frequency.

To determine which phonons are scattered by anharmonic thermal vibrations, we analysed the modal contributions to phonon thermal conductivity in each of the three compounds. Figure 4a,b and c show phonon densities of states (PDOSs) of each compound obtained from LD calculations using the same supercells as in the MD simulations. The partial thermal conductivities of each type of layer as a function of frequency are plotted in Fig. 4d,e and f (magnified views of these plots are shown in Fig. S4 of Supplementary Material). Figure 5 shows the frequency dependence of layer thermal conductivities for CoO_2_ layers in each principal direction. Comparison of PDOSs and frequency dependent partial and layer thermal conductivities reveals which phonons are scattered and which remain heat-conductive.

PDOSs of CoO_2_ layers do not exhibit significant differences between the three materials, in that they are all spread over a wide frequency range. PDOSs of Ca_2_CoO_3_ layers are also spread over a wide frequency range, while those of Na layers are confined to below 10 THz. In NaCoO_2_, the partial thermal conductivity of the CoO_2_ layers is high at relatively low frequencies compared with their PDOS. In the *Z* direction, spectral partial thermal conductivities of Na and CoO_2_ layers show similar behaviour at low frequencies, indicating that vibrations in the two layers are strongly coupled. This leads to less scattering within each type of layer also, resulting in high phonon thermal conductivity in the out-of-plane direction.

In contrast to vacancy-free NaCoO_2_, Na layers make almost no contribution to phonon thermal conductivity in Na_0.5_CoO_2_ because the thermal vibrations of Na atoms are strongly anharmonic. However, vibrations of CoO_2_ layers can be seen to still be coupled with those of the Na ions because PDOSs of the two layer types overlap. Plots of spectral layer thermal conductivity in the in-plane directions in Fig. 5 show that accumulated layer thermal conductivities (measured from the areas under the curves) within CoO_2_ layers in Na_0.5_CoO_2_ are about 66% less than those in NaCoO_2_ below 15 THz and about 9% less above 15 THz compared. Thermal conductivity in CoO_2_ layers is thus strongly suppressed at the lower frequencies over which Na atoms mostly vibrate when a high concentration of Na vacancies is introduced. The sum of the accumulated thermal conductivities below and above 15 THz is almost the same as that of the partial thermal conductivities of CoO_2_ and adjacent layers in Table 1 (the differences are due to the different methods used to calculate the thermal conductivities).

The spectral partial thermal conductivity of CoO_2_ layers in Ca_3_Co_4_O_9_ is much lower than that in Na_0.5_CoO_2_ in all three directions. Figure 5 shows that not only are acoustic phonons strongly suppressed, but so also are the optical phonons at higher frequencies. Accumulated layer thermal conductivities within CoO_2_ layers in the in-plane directions are about 81% smaller than those in Na_0.5_CoO_2_ below 15 THz and 40% smaller above 15 THz. This can be explained in terms of the large overlap up to ~30 THz in the PDOSs of CoO_2_ and Ca_2_CoO_3_ layers, indicating that atomic vibrations within these layers interfere with each other over a wide frequency range. The anisotropic and irregular thermal vibrations of atoms in Ca_2_CoO_3_ layers are thus seen to disturb the thermal vibrations of atoms in the CoO_2_ layers over a wider frequency range, suppressing the rate of phonon transport in these layers even more effectively than the Na layers in Na_0.5_CoO_2_.

Another interesting feature revealed by the plots in Fig. 5 is that the peaks in spectral layer thermal conductivities of CoO_2_ layers in Na_0.5_CoO_2_ and Ca_3_Co_4_O_9_ occur at different frequencies, suggesting that different phonon modes may suppress the thermal conductivity in CoO_2_ layers by different amounts. By appropriate manipulation of the non-CoO_2_ layers, e.g., through doping or making the layers thicker, it may be possible to “tune” the interference caused by phonon anharmonicity to the optimal frequency to minimise thermal conductivity. Such design strategies would also be greatly facilitated if the anharmonic vibrations observed during MD simulations could be connected particular phonon modes, e.g., through a combination of lattice dynamics and Boltzmann transport calculations. At present, however, such analysis is prohibitively expensive for these complex systems, especially since decomposing the overall thermal conductivities calculated from perturbed MD into heat capacities, group velocities and relaxation times is a non-trivial task.

In summary, overall, partial and layer thermal conductivities of layered cobaltites Na_*x*_CoO_2_ (*x* = 1.0 and 0.5) and Ca_3_Co_4_O_9_ calculated using perturbed MD confirmed that CoO_2_ layers transport most of the thermal energy in these materials. In Na_0.5_CoO_2_ and Ca_3_Co_4_O_9_, phonon thermal conduction in CoO_2_ layers is suppressed by anharmonicity in the thermal vibrations of cations in their neighbouring layers. Quantitative analysis of the atomic vibrations revealed differences in the anharmonic behaviour of the non-CoO_2_ layers in Ca_3_Co_4_O_9_ to those in Na_*x*_CoO_2_. Scattered phonons and heat-conductive phonons were identified by modal analysis using perturbed MD in conjunction with LD. In Ca_3_Co_4_O_9_, not only acoustic phonons but also optical phonons are strongly suppressed compared with Na_0.5_CoO_2_ because of the incommensurate stacking of alternating layers, resulting in the lowest thermal conductivity of the three systems examined. These insights suggest that thermal conductivities of layered oxides can be lowered further without compromising their electronic properties by manipulating the amount of anharmonicity in the non-CoO_2_ layers. The same method can also be used to examine alternative ways of introducing and manipulating anharmonicity in oxide and non-oxide materials to accelerate the development of high efficiency thermoelectric energy conversion systems.

## Methods

### Model construction

Reported crystal structures determined using x-ray diffraction^31^ and electron diffraction^32^ were taken as the initial crystal structures for α–NaCoO_2_ and γ–Na_0.5_CoO_2_, respectively. To maintain charge neutrality upon introduction of Na^+^ vacancies in Na_0.5_CoO_2_, half the Co^3+^ ions were changed to Co^4+^ ions. The lowest energy configuration of Na, Co^3+^ and Co^4+^ ions was determined by performing systematic static lattice calculations using the General Utility Lattice Program (GULP) code^33^. The positions of Na ions in the optimized structure were found to be in good agreement with results of electron diffraction studies^32,34^, although they are shifted slightly because Coulombic repulsion between Na^+^ and Co^3+/4+^ ions is overestimated in our model on account of the use of formal charges for the ions.

For Ca_3_Co_4_O_9_, the model of composition (Ca_2_CoO_3_)_3/5_CoO_2_ (CCO) reported by Rébora *et al*. was used as the initial crystal structure^15^. The lowest energy configuration of Co^3+^ and Co^4+^ ions was determined by carrying out first-principles calculations using the Vienna Ab initio Simulation Package (VASP)^35^. This configuration was confirmed to be the most stable by performing static lattice calculations using the GULP code^33^. Details are reported elsewhere^28^.

Supercells of Na_*x*_CoO_2_ and CCO were geometrically optimised using GULP before performing MD and LD simulations. Buckingham potential parameters for Na_*x*_CoO_2_ reported by Tada *et al*.^30^, for O^2–^O^2−^ reported by Cherry *et al*.^36^, and for Ca^2+^-O^2-^ reported by Zacate *et al*.^37^ were employed. Supercell sizes used in MD simulations were 31 × 29 × 31 Å^3^, 30 × 29 × 34 Å^3^ and 25 × 28 × 44 Å^3^ for NaCoO_2_, Na_0.5_CoO_2_ and CCO, respectively.

### Analysis of atomic vibrations

To calculate the lattice constants of the cobaltites at 300 K, MD simulations were carried out in the NPT ensemble for 100 ps with a time step of 1 fs using the Large-scale Atomic/Molecular Massively Parallel Simulator (LAMMPS) code^38^. Next, structural relaxation in the NVT ensemble was performed for an initial 100 ps using temperature scaling and 300 ps using the Nosé-Hoover thermostat to ensure the atoms had reached thermal equilibrium.

Atom positions were recorded during the main production run of 300 ps following structural relaxation. Plots of vibrational density, which follows the curvature of the potential energy hypersurfaces around each atom, were generated by superimposing a Gaussian function on the accumulated atom positions using two- or three-dimensional grids with a mesh size of 0.02 Å. Second derivatives of the density plot intensities (which are proportional to the force constant) at each point were also calculated in each direction using a central difference method. The vibrational centre of each atom was taken to be the grid point whose vibrational density was highest among the points over which the atom vibrated. We used the Gaussian function $G(x,y,\sigma )=\frac{1}{2\pi {\sigma }^{2}}\exp (\,\,-\,\frac{{x}^{2}+{y}^{2}}{2{\sigma }^{2}})$ for two-dimensional density plots, and $G(x,y,\sigma )=\frac{1}{{(2\pi )}^{3/2}{\sigma }^{2}}\exp (\,\,-\,\frac{{x}^{2}+{y}^{2}+{z}^{2}}{2{\sigma }^{2}})$ for three-dimensional density plots. Before calculating second derivatives, the natural logarithm of each intensity curve was calculated, as the Gaussian function contains an exponential term. Average second derivatives and standard deviations were calculated from the values of all ions of the same element within a single MD run. The number of ions was 720 and 360 for Na in NaCoO_2_ and Na_0.5_CoO_2_, respectively, and 480 for Ca in CCO.

### Perturbed molecular dynamics

Phonon thermal conductivities were calculated using the perturbed MD method^22^. The LAMMPS code was modified for this purpose. In this method, phonon thermal conductivity in the *X* direction is calculated according to

1 $$\kappa =\frac{1}{{F}_{{\rm{e}}{\rm{x}}{\rm{t}}}T}\mathop{{\rm{l}}{\rm{i}}{\rm{m}}}\limits_{t\to {\rm{\infty }}}{\langle {J}_{X}\rangle }_{t},$$

where *F*_ext_ is the magnitude of the perturbation in the *X* direction, *T* is temperature and *J*_*X*_ is the heat flux in the *X* direction. The heat flux, *J*_*X*_, is slightly enhanced by the perturbation and proportional to the magnitude of the perturbation within the linear response regime. This cancels out the 1/*F*_ext_ term so that the calculated phonon thermal conductivity is independent of *F*_ext_. According to Irving and Kirkwood^39^, the microscopic heat flux **J** is given by the summation of atomic contributions **J**_*i*_,

2 $${\bf{J}}=\sum _{i}{{\bf{J}}}_{i}=\sum _{i}\frac{1}{2V}[\{{m}_{i}{{{\bf{v}}}_{i}}^{2}{\bf{I}}+\sum _{j}{\varphi }_{ij}{\bf{I}}\}{{\bf{v}}}_{i}-\sum _{j}({{\bf{F}}}_{ij}\cdot {{\bf{v}}}_{i}){{\bf{r}}}_{ij}],$$

where *V* is the volume of the supercell, *m*_*i*_ is the mass of atom *i*, **v**_*i*_ is the velocity of atom *i*, *ϕ*_*ij*_ is the internal energy resulting from interaction between atoms *i* and *j*, **I** is a unit tensor of second rank, and **F**_*ij*_ is the force exerted by atom *j* on atom *i*. The overall thermal conductivity *κ* is given by the sum of atomic thermal conductivities *κ*_*i*_ according to

3 $$\kappa =\sum _{i}{\kappa }_{i}\,=\,\sum _{i}\frac{1}{{F}_{{\rm{e}}{\rm{x}}{\rm{t}}}T}\mathop{{\rm{l}}{\rm{i}}{\rm{m}}}\limits_{t\to {\rm{\infty }}}{\langle {J}_{i,X}\rangle }_{t},$$

By summing heat fluxes over atoms in each layer separately, each layer’s contribution to the overall thermal conductivity can be evaluated. However, the partial thermal conductivities of CoO_2_ layers cannot be simply compared between different cobaltites because atomic contributions may differ depending on the volume of the supercell (or volume ratio between the two types of layer) which is used in eq. (2). In this case, each partial thermal conductivity needs to be scaled by the ratio of layer thicknesses according to

4 $$\kappa {^{\prime} }_{{{\rm{C}}{\rm{o}}{\rm{O}}}_{2}}=\frac{{d}_{{{\rm{C}}{\rm{o}}{\rm{O}}}_{2}}+{d}_{{\rm{a}}{\rm{d}}{\rm{j}}}}{{d}_{{{\rm{C}}{\rm{o}}{\rm{O}}}_{2}}}{\kappa }_{{{\rm{C}}{\rm{o}}{\rm{O}}}_{2}}$$

and

5 $$\kappa {^{\prime} }_{{\rm{a}}{\rm{d}}{\rm{j}}}=\frac{{d}_{{{\rm{C}}{\rm{o}}{\rm{O}}}_{2}}+{d}_{{\rm{a}}{\rm{d}}{\rm{j}}}}{{d}_{{\rm{a}}{\rm{d}}{\rm{j}}}}{\kappa }_{{\rm{a}}{\rm{d}}{\rm{j}}},$$

where ${d}_{{{\rm{CoO}}}_{2}}$ and ${d}_{{\rm{adj}}}$ are the thicknesses of a CoO_2_ layer and an adjacent layer in a given structure, and we refer to $\kappa {\text{'}}_{{{\rm{CoO}}}_{2}}$ and $\kappa {\text{'}}_{{\rm{adj}}}$ as layer thermal conductivities. The thickness of each layer is defined such that the boundary between two layers is midway between the average position of O ions in a CoO_2_ layer and the average position of Na or Ca ions in an adjacent layer in the *Z* direction.

### Thermal conductivity calculations

After achieving thermal equilibrium, perturbed MD simulations were carried out for 1.1 ns. The first 0.1 ns was the time needed for energy fluctuations caused by the perturbation to relax. The average heat fluxes of all constituent atoms were calculated using the last 1.0 ns of data, from which phonon thermal conductivities were derived. More than four different simulations were performed with perturbations of different magnitude within the linear response regime for each cobaltite to obtain an average thermal conductivity and calculate its standard deviation. The atomic thermal conductivities in Fig. 1 were standardised by multiplying by the cross-sectional areas of their supercells perpendicular to the direction of interest in order to facilitate comparison of maps of different sized systems.

### Modal analysis

According to the Green-Kubo modal analysis method developed by Lv and Henry^40^, we can project atomic trajectories from MD onto the modes from LD. The modal contributions to the velocity of each atom, ${{\bf{v}}}_{i}(n)$, can be obtained from the time derivative of the amplitude of normal mode, $\dot{X}(n)$, as

6 $${{\bf{v}}}_{i}=\sum _{n}{{\bf{v}}}_{i}(n)=\sum _{n}\frac{1}{\sqrt{{m}_{i}}}{{\bf{p}}}_{i}(n)\dot{X}(n)$$

where ${{\bf{p}}}_{i}(n)$ is the eigenvector of atom *i* in mode *n*^40,41^. Thus we can obtain modal contributions **J**(*n*) to the microscopic heat flux such that

7 $${\bf{J}}=\sum _{n}{\bf{J}}(n)=\sum _{n}\sum _{i}{{\bf{J}}}_{i}(n)=\sum _{n}\sum _{i}\frac{1}{2V}[\{{m}_{i}{{{\bf{v}}}_{i}}^{2}{\bf{I}}+\sum _{j}{\varphi }_{ij}{\bf{I}}\}{{\bf{v}}}_{i}(n)-\sum _{j}({{\bf{F}}}_{ij}\cdot {{\bf{v}}}_{i}(n)){{\bf{r}}}_{ij}]$$

where **J**_*i*_(*n*) are atomic modal contributions to this flux. In this study, we substituted eq. (7) into eq. (1) instead of using the Green-Kubo expression. With this method, modal or frequency dependencies of overall, atomic, partial, and layer thermal conductivities can be analysed simply from the time average of modal contributions to the heat flux.

LD calculations were performed to obtain normal mode eigenvalues (phonon frequencies), eigenvectors at the gamma point and phonon densities of states of the supercells, using the Phonopy code^42^. The eigenvectors were then read into MD simulations, and calculations of modal thermal conductivity were carried out with one perturbation for each cobaltite system. The calculation procedure used was the same as that described under *Thermal conductivity calculations* except that the time under perturbation was set to 5.1 ns instead of 1.1 ns to improve the statistical accuracy. Further details about the method used to calculate thermal conductivities are given in section S1 of Supplementary Material, and the method of assigning of anharmonic vibrations in CCO to particular phonon modes in section S2.

## Electronic supplementary material

Supplementary Material
