# Supplementary material for: Quantifying Anharmonic Vibrations in Thermoelectric Layered Cobaltites and Their Role in Suppressing Thermal Conductivity
Source: Sci Rep. 2018 Jul 24;8:11152. doi: 10.1038/s41598-018-29259-z (PMC6057949; doi:10.1038/s41598-018-29259-z)
Supplement: Supplementary file 1 — Supplementary Material [file 41598_2018_29259_MOESM1_ESM.pdf]

Supplementary Material for

Quantifying Anharmonic Vibrations in Thermoelectric Layered Cobaltites and Their Role in Suppressing Thermal Conductivity

Susumu Fujii[1], Masato Yoshiya[1,2*] and Craig A. J. Fisher[2]

[1]Department of Adaptive Machine Systems, Osaka University, 2-1 Yamadaoka, Suita, Osaka 565-0871, Japan

[2]Nanostructures Research Laboratory, Japan Fine Ceramics Center, 2-4-1 Mutsuno, Atsuta, Nagoya 456-8587, Japan

*e-mail: yoshiya@ams.eng.osaka-u.ac.jp

S1. Details of thermal conductivity calculations from perturbed molecular dynamics (MD)

As outlineded in the *Methods* section of the paper, in perturbed MD a small net force is exerted on each of the atoms which increases the heat flux in the supercell in a specific direction. The heat flux is proportional to the magnitude of the perturbation force F_{ext} when within the linear response regime. For example, Figure S1 shows the calculated phonon thermal conductivity and the standard deviation in instantaneous thermal conductivity of $NaCoO_2$ as a function of log(F_{ext}) for a pertubation applied in the X direction at 300 K. The phonon thermal conductivity was calculated by averaging the heat fluxes of all constituent atoms at each time step over a period of 1.0 ns, with the standard deviation in the instantaneous thermal conductivity. Up to a certain perturbation, the logarithmic standard deviation decreases linearly with log(F_{ext}) and the average thermal conductivity gradually converges to a constant value as the signal-to-noise ratio increases. However, when $F_{ext} > 6.0\times10^6$ m^{-1}, phonon thermal conductivities and the standard deviations in instantaneous thermal conducitvities suddenly jump to much higher values. This indicates that above 6.0×10^6 m^{-1} the response of the system to the perturbation is no longer in the linear response regime, and thus the calculated phonon thermal conductivity has no physical meaning [1]. To obtain phonon thermal conductivities with good statistical accuracy using perturbed MD, it is thus necessary to perform calculations using a force close to but below that of the linear response limit. In this study in the case of $NaCoO_2$ we determined the final value of phonon thermal conductivity by averaging calculation results with perturbations of seven different magnitudes below 6.0×10^6 m^{-1} in the X direction.

Figure S1 | Phonon thermal conductivity in $NaCoO_2$ in the *X* direction at 300 K as a function of the magnitude of the perturbation, F_{ext}. (a) Average thermal conductivity. **(b)** Standard deviation in instantaneous thermal conductivities. Note that the standard deviations are from instanteneous thermal conductivities during a single run and do not correspond to the uncertainties in thermal conductivity values reported in Table 1.

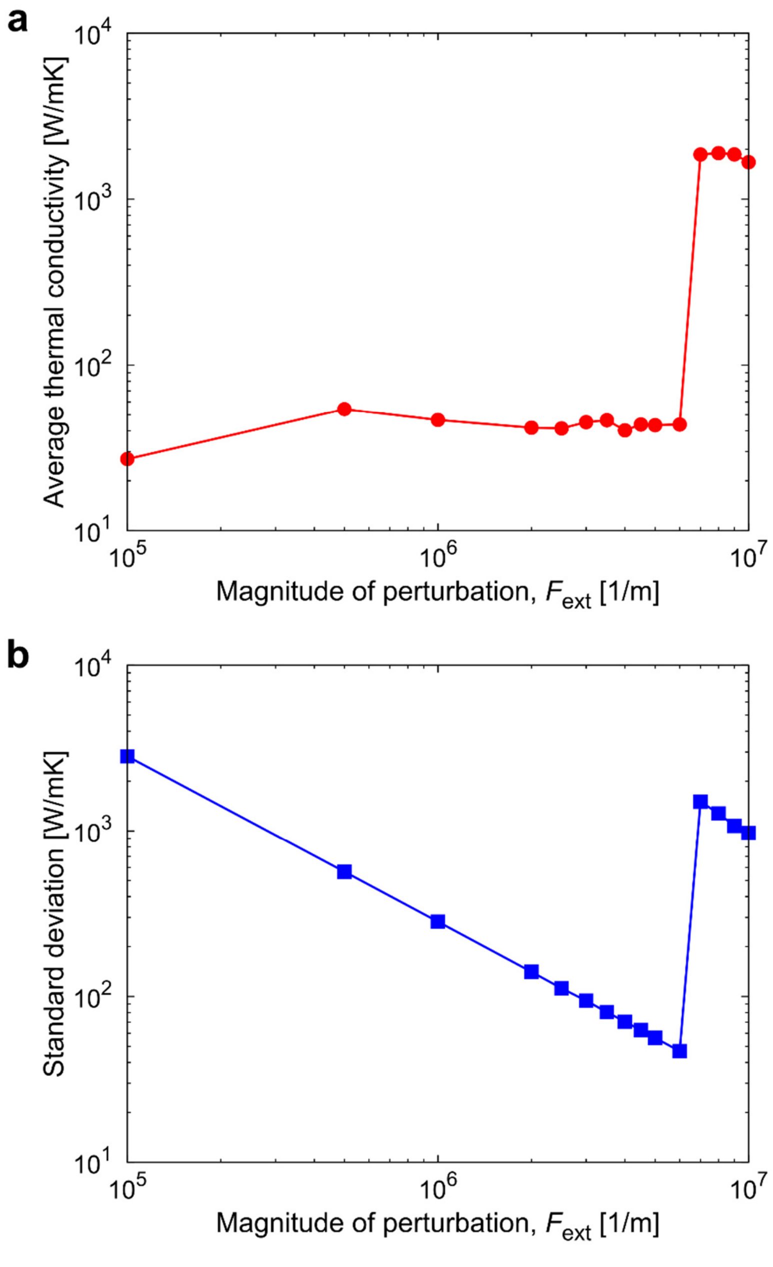

Figure S2 | Plots of vibrational densities of Ca ions in $Ca_3Co_4O_9$. (a), Density plot of atom vibrational motion within a single Ca plane in one Ca_2CoO_3 layer of $Ca_3Co_4O_9$. **(b,c,d,e)**, Examples of density plot isosurfaces of a single Ca ion in $Ca_3Co_4O_9$.

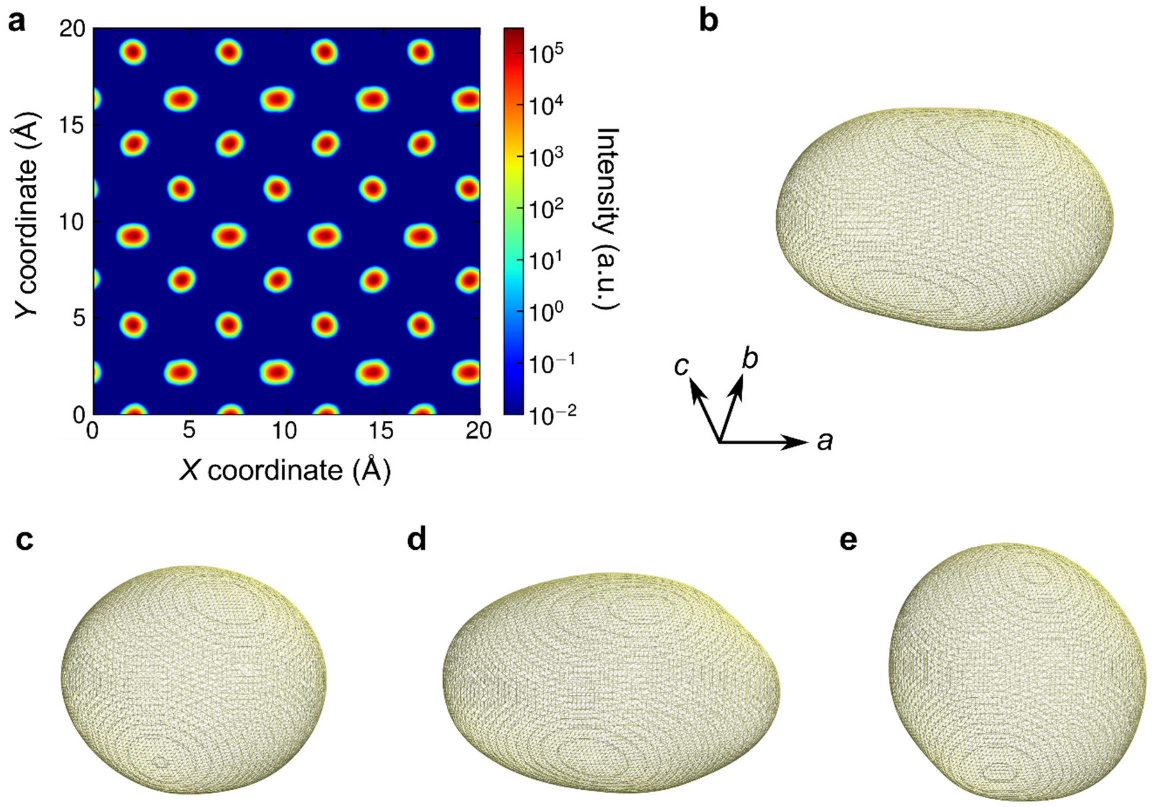

S2. Phonon modes corresponding to anharmonic vibrations in $Ca_3Co_4O_9$

Linking atomic vibrations observed in MD with specific phonon modes calculated by lattice dynamics (LD) can provide greater insight into the thermal conduction mechanisms in complex materials. Figure 3c of the main manuscript shows that the second derivative of vibrational motion of Ca ions in $Ca_3Co_4O_9$ during MD runs is highly asymmetric and its average is dampened in the negative *X* direction, indicating a decrease in the force constant and hence potential energy differential the further an atom is from its vibrational centre. This means that restorative forces exerted on Ca ions (as well as their potential energies) do not increase much when Ca ions are displaced in the negative *X* direction. The specific phonon modes in $Ca_3Co_4O_9$ involved in this phenomenon can be identified by analysing the lattice vibrations in three dimensions. For this we perfomed LD calculations using Phonopy [2] of a single unit cell of the 5/3 approximant to $Ca_3Co_4O_9$, proposed by Rébola et al. [3], i.e., $(Ca_2CoO_3)_{3/5}CoO_2$, using the same Buckingham potential parameters as for the perturbed MD calculations described in the *Methods* section of the paper.

Figure S3a shows the resulting phonon dispersion curves, which are in good agreement with those calculated by Rébola et al. [4] using the GGA+U method. Between the critical points Γ and Z, there are phonons whose frequencies are close to 0, which means that the potential energy surface is almost flat in these directions, i.e., no net forces are exerted on atoms displaced in this direction, and the layers are able to "slosh" back and forth independently of the CoO_2 layers. Atomic vibrations for one such mode are illustrated in Fig. S3b. In this example, lattice vibrations are localised in the Ca_2CoO_3 layer and all its atoms vibrate in the *X* direction with a frequency of 0.6 THz. These localised, low-frequency "floppy" modes result in strongly anharmonic vibrations that are poorly coupled to vibrations in the CoO_2 layers in this system. Although the phonon dispersion curves show small imaginary modes near the Γ point in these zero K calculations (which apparently disappear at the finite temperatures examined by the MD simulations as the structure remains stable), the otherwise good agreement with the DFT results indicates that the observed anharmonic vibrations are an intrinsic feature of the layered CCO material, and not an artefact arising from use of a classical force field.

Figure S3 | Phonon dispersion curves and atomic vibrations for a specific phonon mode. (a) Phonon dispersion curves of the 5/3 approximant to $Ca_3Co_4O_9$ proposed by Rébola et al. [3,4], viz. $(Ca_2CoO_3)_{3/5}CoO_2$, from lattice dynamics calculations using Buckingham potential parameters. **(b)** Atomic vibrations corresponding to the specific phonon mode with frequency of 0.6 THz indicated by the yellow circle in **a**. Light blue, dark blue and red balls represent Ca, Co and O ions, respectively. The arrows represent the relative amplitudes of the atomic vibrations.

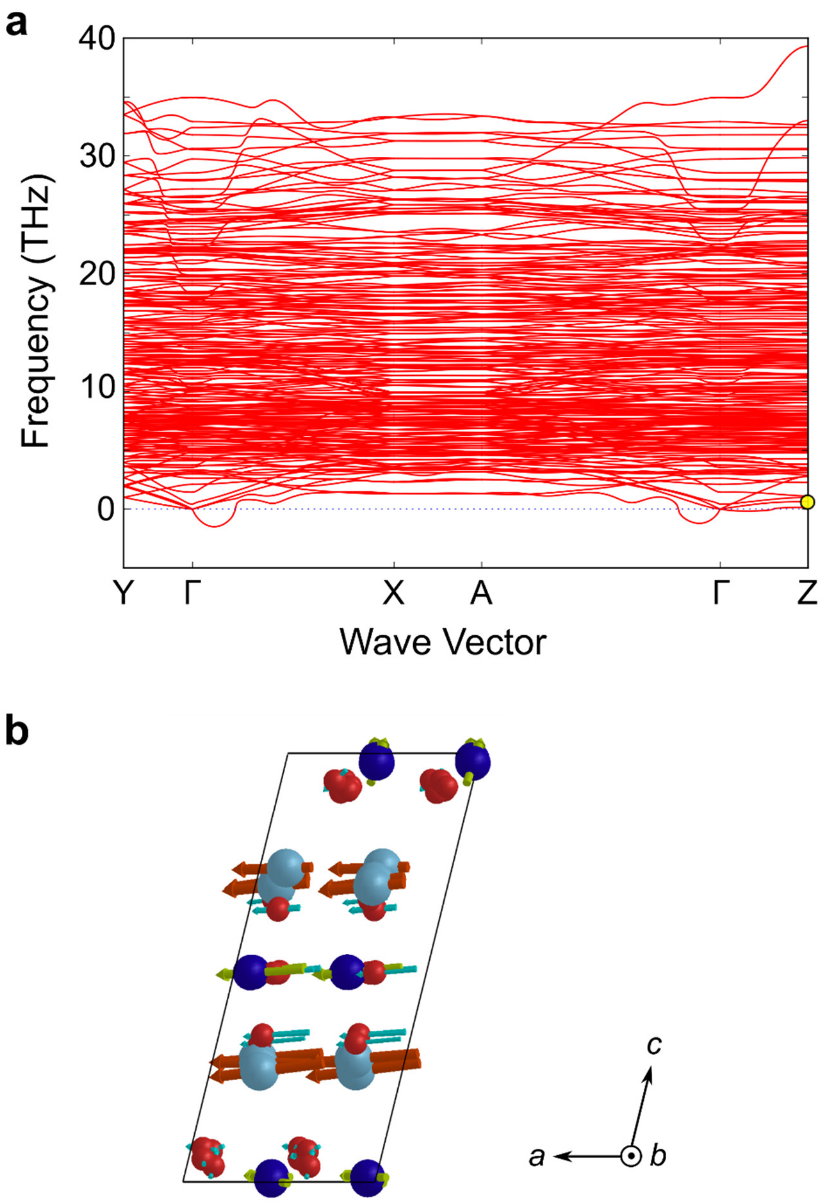

Figure S4 | Magnified views of spectral partial thermal conductivity plots at 300 K. a,**b**,**c**, Frequency dependence of partial thermal conductivity of each type of layer in **(a)** $NaCoO_2$, **(b)** $Na_{0.5}CoO_2$ and **(c)** $Ca_3Co_4O_9$ for in-plane (*X* and *Y*) and out-of-plane (*Z*) directions.

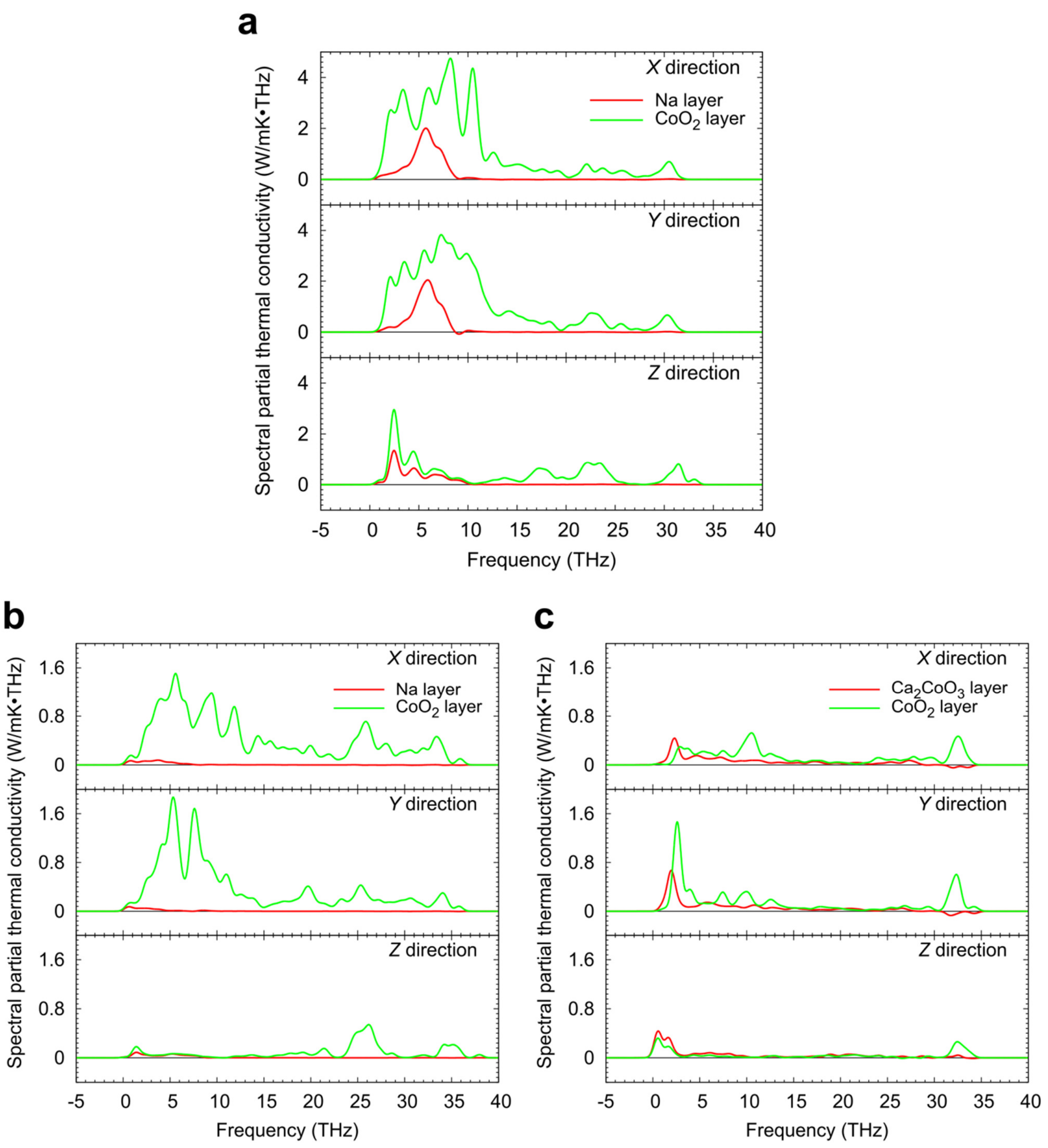

References

[1] Yoshiya, M. *et al.* Perturbed molecular dynamics for calculating thermal conductivity of zirconia. *Mol. Simul.* **30,** 953-961 (2004)

[2] Togo, A. & Tanaka, I. First principles phonon calculations in materials science. *Scripta Mater.* **108**, 1-5 (2015)

[3] Rébola, A. *et al.* First-principles study of the atomic and electronic structures of misfit-layered calcium cobaltite $(Ca_2CoO_3)(CoO_2)_{1.62}$ using rational approximants. *Phys. Rev. B* **85**, 155132 (2012)

[4] Rébola, A. *et al.* Phonon and thermal transport properties of the misfit-layered oxide thermoelectric $Ca_3Co_4O_9$ from first principles. *Appl. Phys. Lett.* **104**, 251910 (2014)
